# Clinical outcomes with vutrisiran in transthyretin amyloidosis: a systematic review and meta-analysis of randomized trials

**DOI:** 10.21542/gcsp.2026.25

**Published:** 2026-06-30

**Authors:** Faizan Ahmed, Faseeh Haider, Ayesha Zulfiqar, Tooba Nihal, Maliha Khalid, Areej Dar, Saman Rauf, Noor ul Ain Saleem, Hassan Farooq, Haris Bin Tahir, Ramsha Ali, Haider Hussain Shah, Swapnil Patel, Mohammad Hossain, Fawaz Alenezi

**Affiliations:** 1 Department of Medicine, Jersey Shore University Medical Centre, Hackensack Meridian Health, Neptune, NJ, USA; 2 Allama Iqbal Medical College, Lahore, Pakistan; 3 Dow University of Health and Sciences, Karachi, Pakistan; 4 Jinnah Sindh Medical University, Karachi, Pakistan; 5 Shaikh Khalifa Bin Zayed Al-Nahyan Medical and Dental College, Lahore, Pakistan; 6 Department of Medicine, Fatima Jinnah Medical University, Lahore, Pakistan; 7 FMH College of Medicine and Dentistry, Lahore, Pakistan; 8 Ameer ud Din Medical College, Lahore, Pakistan; 9 Lahore General Hospital, Lahore, Pakistan; 10 People University of Medical and Health Sciences SBA, Nawabshah, Pakistan; 11 Bayhealth Hospital, Kent Campus, Dover, DE, USA; 12 Division of Cardiology, Department of Medicine, Duke University School of Medicine, Durham, NC, USA

## Abstract

**Background:** Transthyretin amyloidosis (ATTR) is a progressive disease that causes a restrictive cardiomyopathy. Vutrisiran, a subcutaneous RNA interference (RNAi) therapy, is an approved treatment. This systematic review and meta-analysis evaluates its efficacy and safety with respect to transthyretin (TTR) reduction, functional capacity, quality of life, mortality, and adverse events.

**Methods:** We identified 1,032 records, of which three randomized controlled trials—HELIOS-A, HELIOS-B, and a Phase 1 study—comprising 976 participants (508 vutrisiran; 468 comparator) met the inclusion criteria. Comparator participants received placebo, patisiran (an active reference comparator in HELIOS-A), or external placebo from the APOLLO trial. Outcomes assessed were TTR reduction, functional capacity, quality of life, mortality, and adverse events, pooled using random-effects models reporting mean differences and risk ratios.

**Results:** Vutrisiran achieved a rapid, durable TTR reduction of up to 97% in healthy volunteers at the highest dose, and a sustained steady-state reduction in the HELIOS-A and HELIOS-B trials. QoL outcomes showed a protective effect of vutrisiran, with slowed deterioration in the intervention group. Functional outcomes (10-MWT, 6-MWT) suggested slower decline in mobility and functional capacity. Mortality showed a non-significant reduction (RR 0.51; *p* = 0.29; I^2^ = 62%), with most deaths considered unrelated to treatment. The safety analysis showed fewer falls (RR 0.62; *p* = 0.001; I^2^ = 0%) but no significant difference in overall adverse events (AEs) (RR 1.01; *p* = 0.76) or serious AEs (RR 0.82; *p* = 0.23). A sensitivity analysis supported the adverse-event findings.

**Conclusions:** Vutrisiran consistently suppressed TTR and showed signals of benefit in quality of life, function, and mortality, though most of these outcomes did not reach statistical significance. It reduced fall risk without increasing adverse events, indicating a favourable safety profile. Larger, long-term RCTs are needed to confirm survival and functional benefits.

## Introduction

Cardiac transthyretin amyloidosis (ATTR-CM), caused by the deposition of misfolded transthyretin in the myocardium, is increasingly diagnosed in older adults and leads to restrictive cardiomyopathy, conduction defects, and progressive heart failure^1,2^. Improvements in non-invasive diagnostic techniques^3^, along with heightened awareness, have increased detection rates^4^ and underscored the urgent need for disease-modifying therapies. Current management has centered on symptomatic treatment and TTR tetramer stabilizers such as tafamidis, which reduce mortality and hospitalizations^5^. However, significant residual morbidity remains, particularly given the progressive nature of ATTR-CM.

RNA interference (RNAi) offers a targeted mechanism to suppress hepatic TTR synthesis^6^. Vutrisiran, an N-acetyl galactosamine-conjugated small interfering RNA (siRNA) administered subcutaneously every three months, provides sustained TTR knockdown with a more convenient dosing regimen compared to earlier agents.

The HELIOS-B Phase III randomized, double-blind, placebo-controlled trial in patients with hereditary or wild-type ATTR-CM^7^ found that vutrisiran significantly reduced the composite of all-cause mortality and recurrent cardiovascular events. Secondary endpoints—including 6-minute walk distance, Kansas City Cardiomyopathy Questionnaire scores, NYHA class, and NT-proBNP levels—also demonstrated clinically meaningful improvements. Safety findings were favourable, with similar rates of adverse and serious adverse events between vutrisiran and placebo.

However, important questions remain regarding the efficacy and safety of vutrisiran across different ATTR subtypes (hereditary vs. wild-type), disease stages (early vs. advanced), and the influence of concomitant tafamidis use, as well as its long-term safety profile and its comparative effectiveness against other TTR-targeted therapies. We therefore conducted a systematic review and meta-analysis of randomized controlled trials evaluating the efficacy and safety of vutrisiran in ATTR. The primary aim was to estimate pooled effects on all-cause mortality, functional capacity, quality of life, and transthyretin (TTR) reduction, alongside a comprehensive assessment of safety.

## Methods

### Study design and protocol registration

The protocol for this meta-analysis was registered with the International Prospective Register of Systematic Reviews (PROSPERO; ID: CRD420251109407). The review was conducted in accordance with the 2020 update of the Preferred Reporting Items for Systematic Reviews and Meta-Analyses (PRISMA) guidelines^8^. The PICO (Population, Intervention, Comparison, Outcome) framework was used to structure the research question and the eligibility criteria.

### Data sources and search strategy

An electronic search of PubMed, Scopus, ClinicalTrials.gov, Embase, ScienceDirect, and Cochrane was conducted from inception to 2025, without language restrictions. The search used the following keywords: (“Cardiac Amyloidosis” OR “amyloid cardiomyopathy” OR “transthyretin amyloidosis” OR “ATTR amyloidosis” OR “ATTR-CM” OR “light chain amyloidosis” OR “AL amyloidosis”) AND (“Vutrisiran” OR “ALN-TTRSC02” OR “Amvuttra” OR “RNA interference therapy” OR “TTR silencer” OR “TTR siRNA” OR “transthyretin silencing”). The reference lists of retrieved trials, previous meta-analyses, and review articles were manually screened to identify additional relevant studies. The detailed search strategy is provided in Table S1.

### Study selection

We included only randomized controlled trials (RCTs) in this review. Articles retrieved from the literature search were imported into Rayyan.ai, where duplicates were identified and removed^9^. Two independent reviewers screened the remaining records by title and abstract, followed by full-text review to confirm relevance, and only those meeting the predefined eligibility criteria were included. Narrative and systematic reviews, posters, conference abstracts, letters to the editor, and other ineligible articles were screened for references to additional potential RCTs.Eligibility was defined according to the PICO framework:

• **P (Population):** Adults diagnosed with transthyretin amyloidosis (ATTR), including hereditary and wild-type cardiomyopathy.
• **I (Intervention):** Vutrisiran (any dose or regimen).
• **C (Comparator):** Placebo or an active comparator (patisiran).
• **O (Outcomes):** Efficacy outcomes (TTR reduction, walk-test distance, mortality, quality of life) and safety outcomes (incidence of adverse events).

Studies that did not meet these criteria or were not published in English were excluded.

### Outcomes

The primary efficacy outcomes were transthyretin (TTR) reduction, functional capacity (e.g., 6-minute walk test), all-cause mortality, and health-related quality of life. Safety outcomes were the risk of falls, incidence of any adverse event (AE), any serious AE, any severe AE, and the risk of diarrhea.

### Data extraction and quality assessment

Data extraction was performed independently by two reviewers to minimize bias, with discrepancies resolved through discussion or, where needed, consultation with a third reviewer. Extracted data were recorded in a standardized Excel spreadsheet to facilitate consistent comparison and synthesis across studies. The quality of included trials was assessed using the Cochrane Risk of Bias 2 (RoB 2) tool for randomized controlled trials^10^. RoB 2 evaluates five domains: bias arising from the randomization process, deviations from intended interventions, missing outcome data, measurement of the outcome, and selection of the reported result.

### Statistical analysis

All statistical analyses were conducted using RevMan (version 5.4; Copenhagen: The Nordic Cochrane Centre, The Cochrane Collaboration, 2014). Results from trials were presented as mean differences (MDs), standardized mean differences (SMDs), and risk ratios (RRs) with 95% confidence intervals (CIs) and pooled using a random-effects model. The Mantel-Haenszel method was applied to combine RRs. Forest plots were generated to visually represent pooled results, and funnel plots were constructed to assess publication bias. Sensitivity analyses were performed to explore and address heterogeneity. A *p*-value of less than 0.05 was considered statistically significant for all analyses.

## Results

### Search results

**Figure 1.**
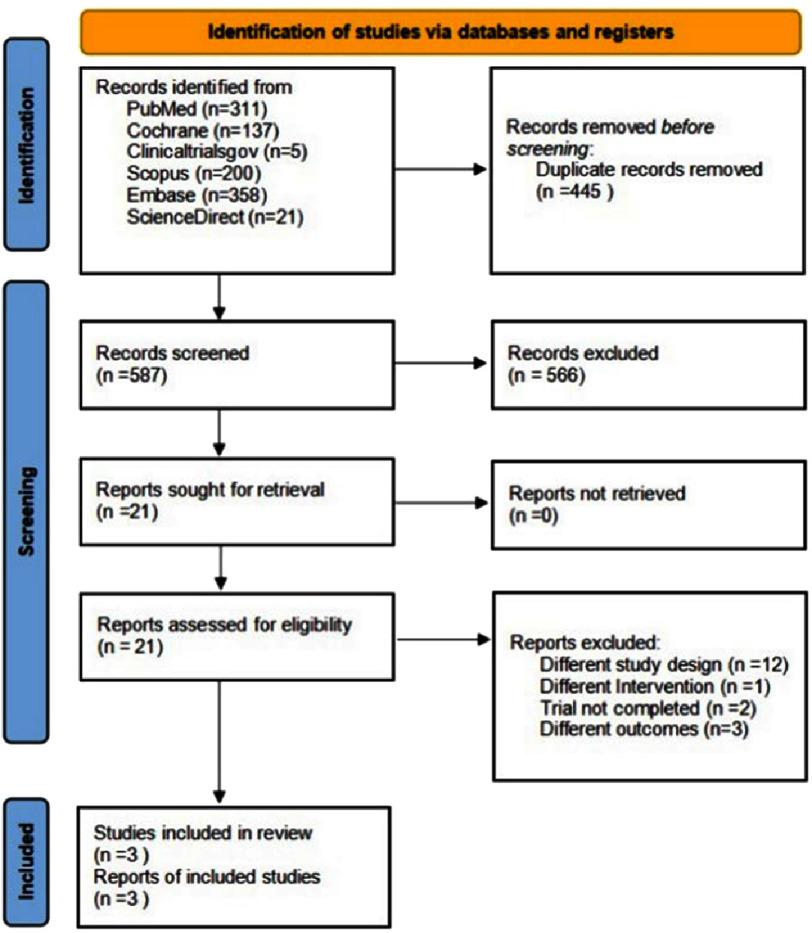
Preferred Reporting Items for Systematic Reviews and Meta-Analyses (PRISMA) flowchart illustrating the study selection process.

A total of 1,032 records were identified through the literature search. After 445 duplicates were removed, 587 records underwent primary screening of titles and abstracts. A further 566 records were excluded, and the full texts of the remaining 21 were retrieved. Secondary screening assessed these full texts for eligibility, and 3 studies meeting the eligibility criteria were included in the systematic review and meta-analysis^7,11,12^. The screening process is detailed in the PRISMA flow diagram (Figure 1).

### Study characteristics

This review includes 3 RCTs published between 2020 and 2025^7,11,12^. One study was conducted in the UK; the remaining two were multicentre^7,11^. A total of 976 participants were included, of whom 896 had ATTR amyloidosis (variant or wild-type, with cardiomyopathy or polyneuropathy) and 80 were healthy volunteers. 508 participants received vutrisiran (the “intervention group”) and 468 received placebo or patisiran (the “control group”). Healthy volunteers received a single subcutaneous dose of vutrisiran (5–300 mg) across dose subgroups, while in the other studies all participants received vutrisiran 25 mg. Tafamidis was used concurrently in only one study and varied across the population: patients on tafamidis at baseline were included in the overall population but excluded from the monotherapy subgroup^7^.

The mean age was 55.9 years and 83.5% of participants were male. Race was reported as 78% White, 11% Asian, and 7% Black or African American. Mean BMI was 24.15 kg/m^2^ among healthy volunteers, and mean mBMI exceeded 1000 in patients with ATTR amyloidosis, reflecting preserved nutritional status^11,12^. NT-proBNP was below 3000 pg/mL in 89.4% of HELIOS-A and 66.8% of HELIOS-B patients, suggesting earlier-stage cardiac involvement^7,11^. Detailed baseline and study characteristics are given in Tables 1 and 2.

**Table 1:** Study characteristics of included studies.

**Study ID**	**Country**	**Study design**	**Intervention**	**Control**	**Primary outcomes**	**Secondary outcomes**	**Number of patients**		
							**Total patients (N)**	**Intervention group (n)**	**Control group (n)**	
**Habtemariam et al. 2020**	United Kingdom	RCT	Vutrisiran (5, 25, 50 mg)	Placebo	Plasma pk parameters, urine pk levels	ADAs, Safety outcomes, Pharmacodynamics	80	60	20	
**Adams et al. 2023**	Multinational	RCT	Vutrisiran 25mg	Placebo	Change in neuropathy impairment measured by a modified Neuropathy Impairment Score Þ7 (mNISÞ7) at month 9	1. Change in QOL by Norfolk QOL-DN 2. Walk test (10-MWT) 3. mNISÞ7 4. Nutritional status by mBMI 5. Disability by [R-ODS] score	199	122	77	
				Patisiran			164	122	42	
**Fontana et al., 2025**	Multicenter	RCT	Vutrisiran 25 mg (Tafamidis at baseline)	Placebo	Mortality from any cause and cv outcomes	1-6 min walk test2-death from any cause3-KCCQ-OS score change	655	326	329	
			Vultisiran 25mg (Monotherapy)	Placebo			395	196	199	

**Notes.**

Abbreviations Used QOL Quality of Life mBMI Modified Body Mass Index NT-proBNP N-terminal pro–B-type Natriuretic Peptide ADAs Anti-Drug Antibodies 10-MWT 10-Meter Walk Test mNIS+7 Modified Neuropathy Impairment Score +7 R-ODS Rasch-built Overall Disability Scale KCCQ-OS Kansas City Cardiomyopathy Questionnaire –Overall Summary score CV outcomes Cardiovascular outcomes SD Standard Deviation N/A Not Available/Not Applicable

**Table 2:** Baseline demographic and clinical characteristics of included studies. Values are shown as intervention/control unless otherwise indicated. “–” denotes an arm with no participants for that comparison.

**Study (n, int/ctrl)**	**Age, yr^1^**	**Male, n (%)**	**mBMI^2^**	**White**	**Asian**	**Black**	**Other**	**NT-proBNP ≤3000^3^, n (%)**
**Habtemariam 2020 (60/20)**	**29.6 (6.82)/29.7 (6.64)**	**27 (45)/11 (55)**	**24.1 (2.85)/24.2 (2.89)**	**31 (51.7)/12 (60)**	**19 (31.7)/5 (25)**	**9 (15)/2 (10)**	**2 (3.3)/2 (10)**	**N/A**
**Adams 2023 (122/77)**	**60 (14.8)/63 (11.1)**	**79 (64.8)/58 (75.3)**	**1057.5 (234)/989.9 (214.2)**	**86 (70.5)/50 (64.9)**	**21 (17.2)/25 (32.5)**	**4 (3.3)/1 (1.3)**	**11 (9.0)/1 (1.3)**	**112 (91.8)/66 (85.7)**
**Adams 2023 —patisiran arm (42)**	**—/60 (8.89)**	**—/27 (64.3)**	**—/N/A**	**—/29 (69.0)**	**—/8 (19.0)**	**—/4 (9.5)**	**—/1 (2.4)**	**—/37 (88.1)**
**Fontana 2025 (326/329)**	**77 (45–85)/76 (46–85)**	**299 (92)/306 (93)**	**N/A**	**277 (85)/275 (84)**	**18 (6)/19 (6)**	**23 (7)/24 (7)**	**8 (2)/10 (3)**	**208 (64)/229 (70)**
**Fontana 2025 —monotherapy (196/199)**	**77.5 (46–85)/76 (53–85)**	**178 (91)/183 (92)**	**N/A**	**169 (86)/169 (85)**	**12 (6)/15 (8)**	**10 (5)/11 (6)**	**5 (3)/4 (2)**	**113 (58)/138 (69)**

**Notes.**

1 Age: mean (SD) for Habtemariam 2020 and Adams 2023; median (range) for Fontana 2025.

2 mBMI = modified BMI (BMI ×serum albumin, g/L). Habtemariam 2020 reports standard BMI in healthy volunteers; values are therefore not directly comparable across studies.

3 Proportion of patients with NT-proBNP ≤ 3,000 ng/L (an indicator of earlier-stage cardiac involvement).

N/A not reported

### Quality appraisal

All included studies were RCTs. Risk-of-bias assessment using the RoB 2 tool indicated that Adams et al., 2023^11^ had low risk of bias across all domains. The other two trials showed low risk in all domains except selection of the reported result (D5): Fontana et al., 2025^7^ was rated as raising some concerns, and Habtemariam et al., 2020^12^ as high risk in that domain. This yields an overall judgement of low risk of bias for HELIOS-A, some concerns for HELIOS-B, and high risk of bias for the Phase 1 healthy-volunteer trial. A detailed assessment is presented in Fig. S1.

### Outcomes

#### Efficacy outcomes

##### Percentage reduction in TTR levels.

Percentage TTR reduction was reported in all three studies. The Phase 1 trial in healthy volunteers showed that a single subcutaneous dose of vutrisiran (5–300 mg) produced a rapid, potent, and sustained reduction in serum TTR. Mean maximum suppression ranged from 57% at the lowest doses to 97% at the highest, and the effect was maintained for at least 90 days post-dose^12^. In HELIOS-A, in patients with hereditary ATTR amyloidosis with polyneuropathy, vutrisiran-treated patients (*n* = 118) dosed every three months achieved a 6.3% greater steady-state TTR reduction than the patisiran control group (*n* = 37) at month 18. These reductions were evident early, remained stable through 18 months, and showed no evidence of waning over time^11^.

In HELIOS-B, which enrolled patients with ATTR amyloidosis with cardiomyopathy, Vutrisiran produced substantial and durable percentage TTR suppression of 79.8% from baseline in monotherapy population, although exact pooled values were not a primary endpoint^7^. Due to major differences in study populations, pooled quantitative analysis was not deemed appropriate.

##### Quality of life (QoL).

HELIOS-A and HELIOS-B reported QoL data, assessed with the Norfolk Quality of Life–Diabetic Neuropathy (QOL-DN) score and the Kansas City Cardiomyopathy Questionnaire–Overall Summary (KCCQ-OS), respectively^7,11^. These covered 574 patients (310 intervention, 264 control). In HELIOS-A, vutrisiran significantly improved the Norfolk QOL-DN score in 53.4% of patients, with improvement by month 9 and stable health status maintained through month 18^11^. In HELIOS-B, the two groups had similar baseline KCCQ-OS scores (70.3 vs 69.9). Both groups in the monotherapy population declined over 30 months, so vutrisiran did not improve QoL in absolute terms; however, the slower rate of decline in the intervention group (−10.8 vs −19.5) suggests a protective effect. Overall, vutrisiran does not appear to improve QoL outright but may attenuate the decline in health status over time in patients with ATTR amyloidosis. A direct quantitative comparison was not performed because the two trials used different QoL instruments.

##### Walk test.

Two of the three included studies reported this outcome, covering 562 patients (308 intervention, 254 control). The 10-metre walk test (10-MWT) assessed gait speed in patients with hereditary ATTR amyloidosis with polyneuropathy in HELIOS-A, and the 6-minute walk test (6-MWT) assessed walk distance in patients with ATTR amyloidosis with cardiomyopathy in HELIOS-B^7,11^. In HELIOS-A, gait speed progressively declined from baseline to months 9 and 18 (−0.133 and −0.264 m/s) in the placebo group, whereas the vutrisiran group showed negligible decline, particularly at month 18, suggesting preserved mobility over this period^11^. In HELIOS-B, both groups declined in 6-MWT distance over 30 months, reflecting progressive functional deterioration; the smaller decline in the monotherapy intervention group (by 32 metres) suggests that vutrisiran better preserved physical function over time^7^. Because the two trials used different functional assessment tools, quantitative pooling was not performed.

##### Mortality.

**Figure 2.**
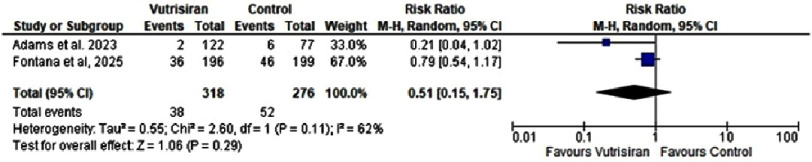
Forest plot of mortality.

All-cause mortality was reported in HELIOS-A and HELIOS-B. Both showed a non-significant reduction in deaths among vutrisiran-treated patients—more pronounced in HELIOS-A (RR 0.21; 95% CI [0.04–1.02]) than in the HELIOS-B monotherapy population (RR 0.79; 95% CI [0.54–1.17]). In the pooled analysis, vutrisiran was associated with a lower risk of death that did not reach statistical significance (RR 0.51; 95% CI [0.15–1.75]; *p* = 0.29), with moderate heterogeneity (I^2^ = 62%). Notably, the two deaths (1.6%) in the HELIOS-A vutrisiran group were attributed to COVID-19 or cardiac disease rather than to treatment^11^. Taken together, these findings suggest a possible mortality benefit that remains unconfirmed. The result is shown in Figure 2.

#### Safety

##### Risk of fall.

**Figure 3.**
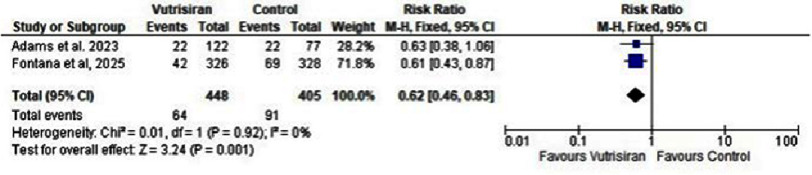
Forest plot of risk of fall.

The two studies (HELIOS-A and HELIOS-B), comprising 853 participants (448 vutrisiran, 405 placebo), recorded the incidence of falls^7,11^. In HELIOS-A, the risk ratio (RR) was 0.63 (95% CI [0.38–1.06]), suggesting a lower risk of falls in the vutrisiran group, though this did not reach statistical significance. In HELIOS-B, the RR was 0.61 (95% CI [0.43–0.87]), a statistically significant reduction in the overall population. The pooled estimate was 0.62 (95% CI [0.46–0.83]; *p* = 0.001), a 38% relative reduction in fall risk, with no heterogeneity across studies (I^2^ = 0%). The result is shown in Figure 3.

##### Any adverse event.

**Figure 4.**
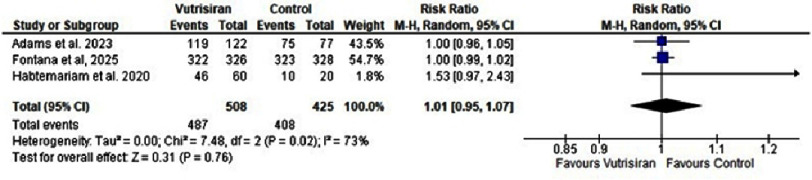
Forest plot of any adverse effect.

The incidence of any adverse event was reported in all three studies. HELIOS-A and HELIOS-B both reported similar adverse-event incidence (mostly mild or moderate) in the intervention and control groups, affecting roughly 97–99% of participants over the treatment period^7,11^. In Habtemariam et al., adverse events—all mild—were reported in 46 participants (77%) in the vutrisiran group and 10 (50%) in the placebo group. The pooled analysis of all three trials (933 participants) showed no significant difference in the risk of any adverse event between the vutrisiran and control groups (RR 1.01; 95% CI [0.95–1.07]; *p* = 0.76), with substantial heterogeneity (I^2^ = 73%). A sensitivity analysis excluding the Habtemariam trial—the main contributor to heterogeneity—yielded nearly identical results (RR 1.00; 95% CI [0.99–1.02]; *p* = 0.74) and reduced heterogeneity to zero (I^2^ = 0%), supporting the robustness of the finding (Fig. S2; Figure 4).

##### Any serious adverse event.

**Figure 5.**
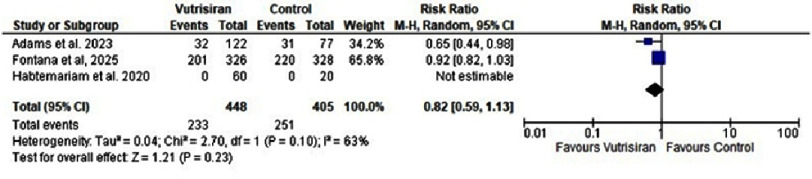
Forest plot of any serious adverse effect.

All three studies reported this outcome. In HELIOS-A, serious adverse events were reported in 32 patients (26.2%) in the vutrisiran group, but only two (1.6%) had serious adverse events considered related to vutrisiran (one dyslipidaemia, one urinary tract infection)^11^. In HELIOS-B, 201 patients (62%) in the vutrisiran group of the overall population reported a serious adverse event, irrespective of tafamidis use^7^. The healthy-volunteer study (Habtemariam et al.) reported no serious adverse events in either arm and was not included in the pooled estimate^12^. Although HELIOS-A reported a statistically significant reduction in risk (RR 0.65; 95% CI [0.44–0.98]), the pooled analysis with HELIOS-B showed no significant difference between the vutrisiran and control groups (RR 0.82; 95% CI [0.59–1.13]; *p* = 0.23). Heterogeneity was moderate (I^2^ = 63%; *p* = 0.10) (Figure 5).

##### Any severe adverse event.

**Figure 6.**
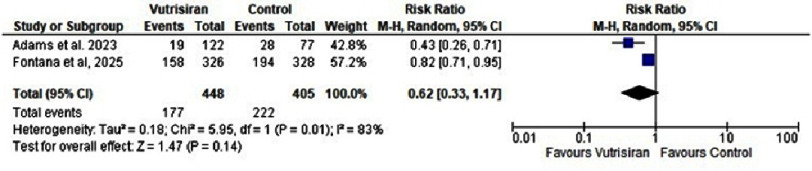
Forest plot of any severe adverse effect.

All three studies were assessed for severe adverse events; the healthy volunteers in the Habtemariam et al. Phase 1 trial reported none. In HELIOS-A, 19 patients (15.6%) in the intervention group experienced severe adverse events, with an RR of 0.43 (95% CI [0.26–0.71]), suggesting a significant 57% reduction. In HELIOS-B, 48% of patients on vutrisiran in the overall population experienced severe adverse events, compared with 59% in the control group^7^. The pooled analysis of the two estimable trials showed no statistically significant difference between vutrisiran and control (RR 0.62; 95% CI [0.33–1.17]; *p* = 0.14), with high heterogeneity (I^2^ = 83%; *p* = 0.01) (Figure 6).

##### Risk of diarrhea.

**Figure 7.**
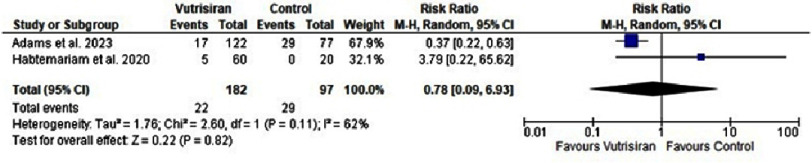
Forest plot of diarrhea.

Two of the three included studies reported diarrhoea as an adverse event. In Habtemariam et al., diarrhoea occurred in 5 healthy volunteers (8.3%) across vutrisiran doses (5–300 mg)^12^. In HELIOS-A, diarrhoea was reported in 14% of patients in the vutrisiran group *versus* 38% in the placebo group (RR 0.37), a significant reduction^11^. The pooled analysis showed no significant association between vutrisiran and diarrhoea risk (RR 0.78; 95% CI [0.09–6.93]; *p* = 0.82), with moderate heterogeneity (I^2^ = 62%) (Figure 7).

## Discussion

This systematic review of clinical-trial evidence supports a sustained biological and functional effect of vutrisiran in transthyretin amyloidosis (ATTR), spanning hereditary neuropathic and cardiac phenotypes. Across study designs and populations, vutrisiran produced significant and durable suppression of circulating TTR—a mechanistic prerequisite for preventing amyloid fibril formation^13^. Functional assessments, including walking speed, walk distance, and patient-reported quality of life, showed a pattern of blunted decline rather than reversal, consistent with the progressive, multisystem course of ATTR. Mortality reductions were not statistically significant, though the direction of effect favoured vutrisiran. The safety profile was characterised by a significant reduction in fall risk without an increase in total, serious, or severe adverse events. Taken together, these findings support vutrisiran as an effective TTR-lowering therapy with good tolerability, and highlight functional stabilisation and fall reduction as clinically meaningful outcomes in ATTR.

The primary pharmacodynamic effect—reduction of circulating TTR—was consistent across the included trials. Persistent TTR suppression is considered central to slowing amyloid deposition and disease progression^11,14^. In HELIOS-A, vutrisiran produced sustained TTR suppression alongside preservation of neuropathy-related functional outcomes and quality-of-life measures^11,14^. Although several pooled efficacy endpoints did not reach statistical significance, the direction of effect was consistent across studies and favoured treatment benefit. This heterogeneity likely reflects differences in trial design, patient populations, and outcome measures rather than inconsistency in the drug’s biological activity. The finding aligns with the model that suppressing amyloidogenic TTR production is the most direct disease-modifying strategy in ATTR. Nevertheless, questions remain about long-term safety, possible off-target effects, and the optimal monitoring of treatment response, particularly in cardiac involvement^15^.

The QoL trends reflect a therapeutic reality of ATTR: stabilisation may be a more meaningful and achievable goal than improvement, particularly in established disease^16^. The stabilisation of Norfolk QOL-DN scores in HELIOS-A echoes earlier observations from APOLLO (patisiran) and NEURO-TTR (inotersen), in which treated patients maintained neuropathy-related quality of life while placebo groups declined^11,17–19^. In HELIOS-B, the deterioration in KCCQ-OS scores appeared attenuated over time. This blunted decline, even where not statistically significant in pooled analysis, carries clinical meaning: in a progressive disease marked by cumulative functional loss, slowing the rate of decline can translate into prolonged independence and reduced care needs. The discrepancies in pooled estimates are attributable to heterogeneity in measurement instruments, disease phenotype, and baseline symptom burden. Mobility endpoints—the 10-metre and 6-minute walk tests—provide complementary information on the preservation of function.

Near-stable gait speed over 18 months in HELIOS-A is notable, given that untreated hereditary ATTR polyneuropathy typically leads to progressive loss of ambulation^6^. Similarly, the reduced deterioration in 6-minute walk distance in HELIOS-B suggests preservation of exercise capacity in cardiac ATTR. These converging findings point to a shared mechanism: by lowering amyloidogenic TTR, the drug may limit further nerve and muscle damage, reduce autonomic instability, and slow cardiomyopathic stiffening—factors that together support mobility. The lack of statistical significance in pooled analysis probably reflects variation in mobility endpoints and in baseline functional reserve between the neuropathic and cardiomyopathic groups.

Mortality findings should be interpreted cautiously. Although pooled mortality analyses were not statistically significant, the effect estimates generally favoured vutrisiran. Relatively short follow-up, low event rates, and variation in disease severity limit firm conclusions about survival benefit. Longer-term studies will be needed to determine whether these trends translate into clinically meaningful mortality reductions^20^.

The reduction in fall risk observed here is both clinically meaningful and mechanistically consistent with vutrisiran’s effects. Falls in ATTR are typically multifactorial, driven by sensory neuropathy, motor weakness, autonomic dysfunction, and impaired balance. By slowing neuropathic progression and potentially preserving proprioception, vutrisiran may act directly on fall risk^7,21^. Its effectiveness in delaying disease progression and improving several outcomes underscores the value of early detection and treatment, since patients with less severe baseline neuropathy showed better scores at 18 months^22^. Importantly, the fall-reduction result showed low heterogeneity across studies, strengthening confidence in this pooled signal and marking falls as a potentially valuable patient-centred endpoint for future ATTR trials.

The high but balanced incidence of adverse events across treatment and control arms likely reflects the underlying frailty of ATTR patients rather than drug toxicity. This is consistent with safety observations for both RNA-targeted and stabiliser therapies, in which most adverse events are mild to moderate and unrelated to the study drug^11^. Vutrisiran’s quarterly subcutaneous dosing likely supports its tolerability by avoiding infusion reactions and reducing treatment burden—an advantage over intravenous therapies that require frequent clinic visits.

The variable picture for serious adverse events—significant in one trial but null in the pooled estimate—mirrors the pattern seen with tafamidis and patisiran, where serious-event rates remain high because of the systemic nature of ATTR. Most such events in these trials were cardiac or systemic decompensations rather than treatment toxicity. The moderate heterogeneity here likely reflects variability in disease stage at entry and in reporting methods rather than any true divergence in the safety signal. Likewise, serious adverse events did not differ significantly between treatment and control in the pooled analysis, despite some trial-specific reductions. Given ATTR’s multisystem nature, such events—typically hospitalisations for cardiac or neurological complications—are expected even with effective disease-modifying therapy^23^.

The absence of a consistent association between vutrisiran and diarrhoea is in keeping with the generally benign gastrointestinal tolerability of RNA interference therapies. The diarrhoea observed most likely reflects the gastrointestinal autonomic neuropathy and amyloid deposition characteristic of ATTR, arising largely independently of treatment. This is consistent with earlier patisiran safety experience, in which diarrhoea was not a treatment-limiting toxicity^24^.

## Limitations

Several limitations should be considered when interpreting these findings. The evidence base is small and clinically diverse: only three trials met inclusion, and they differed substantially in design, population, and outcome measures. Most consequentially, the analysis combined a single-ascending-dose study in healthy volunteers with trials in two distinct disease phenotypes—hereditary ATTR with polyneuropathy (HELIOS-A) and ATTR with cardiomyopathy (HELIOS-B). The healthy-volunteer study contributed only pharmacodynamic and short-term safety data and was judged to be at high risk of bias, while pooling neuropathic and cardiac populations combines groups whose natural history, baseline functional reserve, and outcome instruments are not directly comparable. These differences limit how far any pooled estimate can be generalised to a single ATTR population, and they are the principal source of the statistical heterogeneity observed across endpoints.

This heterogeneity also constrained the analysis itself. For several efficacy outcomes—including TTR reduction, quality of life, and functional capacity—the use of different measurement instruments across trials meant that quantitative pooling was not appropriate, and these outcomes were synthesised narratively rather than meta-analysed. Where pooling was performed, the small number of trials and low event rates produced wide confidence intervals for several estimates, most notably mortality and diarrhoea. Non-significant pooled results should therefore be read as imprecise rather than as evidence of no effect; the analysis was underpowered to detect clinically plausible differences in these outcomes.

Follow-up durations were also relatively short for endpoints that evolve over years, particularly survival, where few events accrued. Finally, and most fundamentally for interpreting the headline finding, TTR reduction is a validated pharmacodynamic marker but a surrogate: the extent to which sustained TTR lowering translates into organ recovery, rather than slowed deterioration, remains incompletely established—especially in patients with a high amyloid burden, in whom existing structural damage may limit the ceiling of clinical benefit. The consistent and substantial TTR suppression demonstrated here is best understood as a mechanistic foundation for benefit rather than as direct evidence of disease reversal.

## Conclusion

Overall, these findings position vutrisiran as an effective and well-tolerated TTR-lowering therapy that may stabilise key functional endpoints and reduce fall risk without an increase in major safety events. Although several pooled efficacy outcomes did not reach statistical significance, the consistent direction of effect across studies points to a potential clinical benefit that longer and larger trials will need to confirm. The durable biological effect, alongside the observed pattern of slowed decline, supports the rationale that sustained TTR reduction acts to modify disease progression. The reduction in fall risk is one of the most clinically relevant findings of this meta-analysis and underscores the value of patient-centred functional outcomes in ATTR management. While survival and quality-of-life benefits require further clarification, particularly with longer follow-up, vutrisiran’s quarterly subcutaneous dosing and favourable tolerability may ease treatment burden and support adherence, with possible downstream effects on long-term outcomes. Trials with harmonised endpoints and extended follow-up will be central to defining the full clinical scope of vutrisiran’s effect across both the cardiac and neuropathic forms of ATTR.

## Declarations

**Data Availability Statement:** All data generated or analyzed during this study are included in the manuscript and its supplementary file.

**Funding Statement:** This research did not receive any grant from funding agencies in the public, commercial or not-for-profit sectors.

**Ethics Approval Statement:** Not applicable. This study is a meta-analysis and did not involve human participants or animal subjects.

**Patient Consent Statement:** Not applicable.

**Permission to Reproduce Material from Other Sources**: Not applicable. No third-party material was reproduced.

## Conflict of Interest Disclosure

The authors declare no conflicts of interest related to this study. All authors have reviewed and approved the final manuscript.
